# Who Underreports Smoking on Birth Records: A Monte Carlo Predictive Model with Validation

**DOI:** 10.1371/journal.pone.0034853

**Published:** 2012-04-24

**Authors:** Thomas G. Land, Anna S. Landau, Susan E. Manning, Jane K. Purtill, Kate Pickett, Lauren Wakschlag, Vanja M. Dukic

**Affiliations:** 1 Massachusetts Department of Public Health, Boston, Massachusetts, United States of America; 2 Department of Applied Mathematics, University of Colorado, Boulder, Colorado, United States of America; 3 Department of Health Sciences, University of York, York, United Kingdom; 4 Northwestern University Feinberg School of Medicine, Chicago, Illinois, United States of America; University of Liverpool, United Kingdom

## Abstract

**Background:**

Research has shown that self-reports of smoking during pregnancy may underestimate true prevalence. However, little is known about which populations have higher rates of underreporting. Availability of more accurate measures of smoking during pregnancy could greatly enhance the usefulness of existing studies on the effects of maternal smoking offspring, especially in those populations where underreporting may lead to underestimation of the impact of smoking during pregnancy.

**Methods and Findings:**

In this paper, we develop a statistical Monte Carlo model to estimate patterns of underreporting of smoking during pregnancy, and apply it to analyze the smoking self-report data from birth certificates in the state of Massachusetts. Our results illustrate non-uniform patterns of underreporting of smoking during pregnancy among different populations. Estimates of likely underreporting of smoking during pregnancy were highest among mothers who were college-educated, married, aged 30 years or older, employed full-time, and planning to breastfeed. The model's findings are validated and compared to an existing underreporting adjustment approach in the Maternal and Infant Smoking Study of East Boston (MISSEB).

**Conclusions:**

The validation results show that when biological assays are not available, the Monte Carlo method proposed can provide a more accurate estimate of the smoking status during pregnancy than self-reports alone. Such methods hold promise for providing a better assessment of the impact of smoking during pregnancy.

## Introduction

In a 2001 report, the Surgeon General of the United States described decades of research into the specific health effects of cigarette smoking on women [1]. The report catalogues a long list of pregnancy complications and adverse birth outcomes associated with smoking during pregnancy but perhaps the most thoroughly documented is that of low birth weight [2]–[5]. The specific mechanism by which cigarette smoking leads to low birth weight is not completely understood. Moreover, there is no single level of cotinine or nicotine that can be uniquely associated with a specific number of cigarettes smoked. Further complicating this picture is evidence that the metabolism of nicotine and cotinine is accelerated during pregnancy [6]–[8]. Two methods of cotinine-based calibration of self-reports have been proposed recently to detect and adjust for underreporting [9], [10]. However, the adjustment methodology proposed by Dukic requires cotinine measures and thus is impractical for large population studies.

In contrast, the most common source of information about smoking during pregnancy are self-reports on birth certificates (BCs). Honein's extensive study of BCs included data from 45 states, New York City, and the District of Columbia [11]. The study concluded that, despite some obvious weaknesses in BC data, this information source is still quite useful in studying the association between maternal risk factors recorded on birth certificates and adverse birth outcomes including birth defects.

Massachusetts BC data shows that the self-reported rate of smoking during pregnancy has dropped every year between 1989 (13.1%) and 2004 (7.2%) [12]. If the prevalence numbers are accurate, a decrease of such magnitude is undeniably good news. However, the vast majority of studies conducted in the U.S. have found significant levels of underreporting [13]–[20].

By understanding how underreporting patterns relate to specific population characteristics, tailored programs could be developed for physicians and hospitals to increase the accuracy of the information gathered. The purpose of this paper is: 1) to develop a statistical model to estimate the likelihood of underreporting of smoking during pregnancy; 2) to identify maternal demographic and socio-economic characteristics associated with estimated underreporting and assess variations in estimated underreporting across birth facilities; and 3) to validate the results of this model on a separate dataset, the Maternal and Infant Smoking Study of East Boston (MISSEB) Study [21], [22], where cotinine-calibrated self reports have been provided [9], [10]. In an effort to ensure transferability of results from one model to the other we focused on data sources with information gathered from Massachusetts residents only.

The ultimate goal of the study is to bridge the gap between population based datasets like BCs and smaller datasets with more bioassay information. By demonstrating that serum cotinine levels are significantly related to profiles of demographic and socioeconomic information developed on BCs, it should open the door to more a more accurate assessment of smoking during pregnancy.

## Methods

Two parallel datasets were analyzed in this study. The first analysis was done on a dataset that included demographic and smoking behavior data from the Massachusetts BRFSS, the largest continuously conducted telephone-based health surveillance system in the world [23], for 1997 through 2004. The second analysis was done on a dataset with similarly coded demographic and smoking behavior data from Massachusetts BC's for singleton live births during the same time period. Both datasets were restricted to women ages 18–44. Sixteen indicator variables were constructed to characterize the main demographics and socio-economic characteristics (age, educational level, marital status, employment status, race, and ethnicity) for 18,533 BRFSS records and 605,095 birth records.

**Figure 1:**
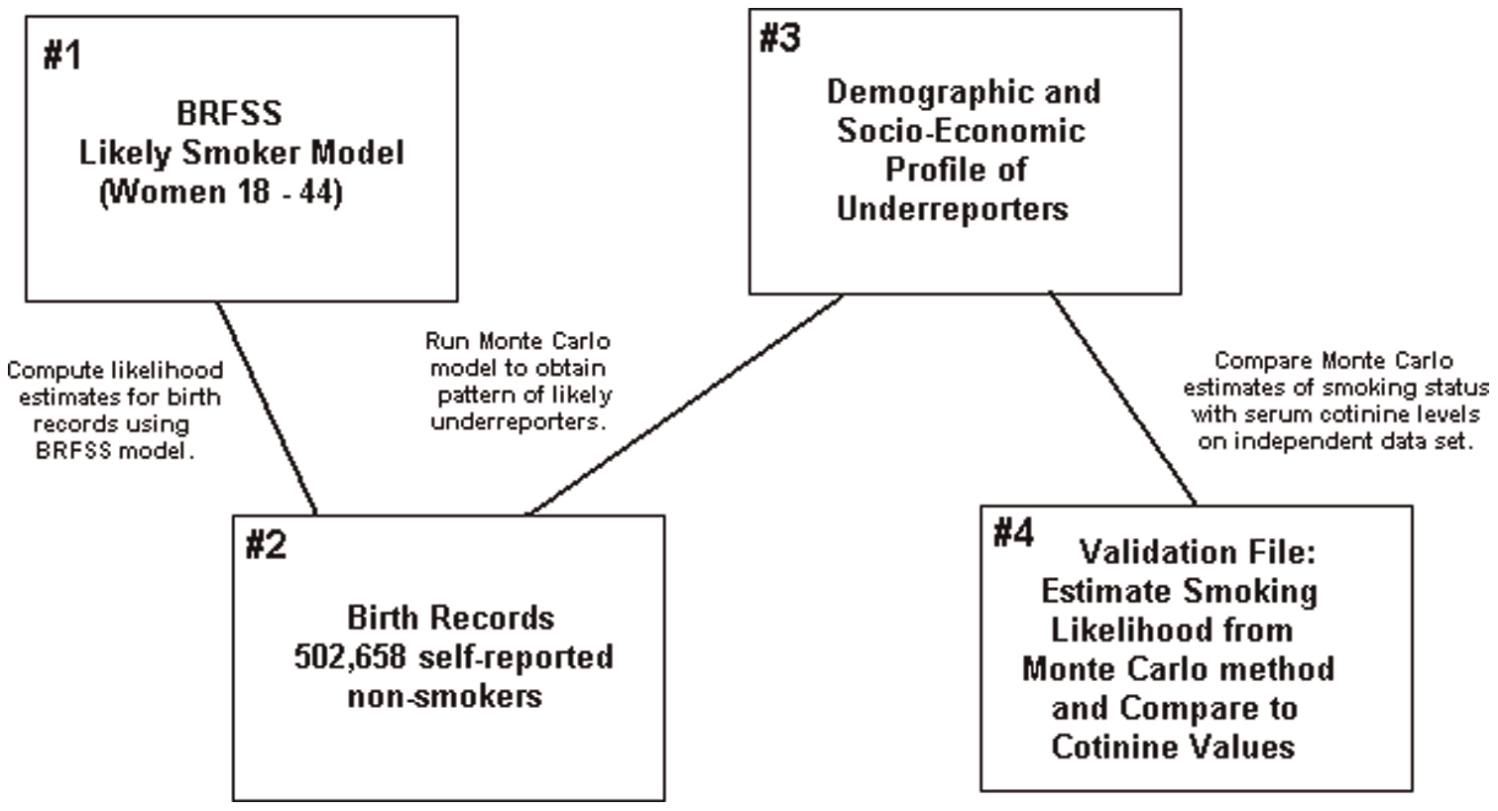
Logic behind the analysis in the paper.

Figure 1 depicts the overarching logic for this study. The brief analytic description below is followed a detailed description of the methodology.

  - Step-1: Data from the Massachusetts Behavioral Risk Factor Surveillance System (BRFSS) collected between 1997 and 2004 was used to compute a logistics regression of likely smoking for Massachusetts women age 18 to 44.
  - Step-2: The BRFFS likely smoker model was used to score 502,658 self-reported non-smokers (i.e., all self-reported non-smokers from Massachusetts birth records between 1997 and 2004).
  - Step-3: A Monte Carlo procedure was run to create a demographic and socio-economic profile of likely underreports based on non-uniform patterns of infant birth weight.
  - Step-4: The demographic and socio-economic profile from the Monte Carlo analysis was used to estimate the smoking likelihood of self-reported non-smokers who also serum cotinine tests available. As a validation, the likelihood estimates and the serum cotinine levels were compared by correlation.

Using BRFSS data, a generalized linear predictive model was developed to estimate current levels of smoking among women ages 18–44 (no restriction was made for pregnancy as this would have excluded over 96% of all records.) The model used the significant subset of the sixteen demographic and socio-economic variables as the main-effect predictors, while a stepwise logistic regression (SAS V9.1) was used to select additional 2-way interactions (inclusion criteria was p<0.001). Data were weighted using FINALWT, a standard BRFSS population weighting variable, as shown in Table 1.

**Table 1:** Adjusted odds ratios for BRFSS likely smoker model (Massachusetts BRFSS: 1997–2004).

Parameter	Primary Term	Interaction Term	Adjusted Odds Ratio	Significance Level
WHITE NON-HISPANIC	X		**1.75**	**p<.001**
HISPANIC	X		**0.67**	**p<.001**
MARRIED	X		**0.49**	**p<.001**
NO H.S. DEGREE	X		**2.49**	**p<.001**
H.S. DEGREE	X		1.12	p = .23 (n.s.)
COLLEGE OR MORE	X		**0.40**	**p<.001**
EMPLOYED	X		**0.57**	**p<.001**
STUDENT	X		**0.41**	**p<.001**
HOMEMAKER	X		**0.72**	**p<.01**
AGE – UNDER 21	X		**0.42**	**p<.001**
WHITE × HISPANIC		X	**0.49**	**p<.001**
WHITE × H.S. DEGREE		X	**1.42**	**p<.001**
HISPANIC × NO DEGREE		X	**0.46**	**p<.001**
HISPANIC × COLLEGE OR MORE		X	**2.50**	**p<.001**
MARRIED × COLLEGE OR MORE		X	**0.78**	**p<.01**
MARRIED × HOMEMAKER		X	**0.50**	**p<.001**
NO DEGREE × HOMEMAKER		X	**1.86**	**p<.001**
AGE – UNDER 21 × EMPLOYED		X	**1.78**	**p<.01**

n.s. =  Non-significant.

Assuming some level of underreporting, a more accurate population estimate of prevalence would require that smoking status for a proportion of mothers be reclassified. Each reclassification, however, would result in a change in the average birth weight difference between the resulting “smoker” and “non-smoker” groups. For example, one might make a random selection of self-reported non-smokers and reclassify them as smokers. In doing that, the average birth weights for the reconstituted groups would almost certainly be different than the means for original groups.

Using the estimated generalized linear predictive model fitted on BRFSS data, predicted likelihood of smoking was computed for each of the 605,095 birth records. In the second tier of our analysis, these likelihood estimates become weights in our Monte Carlo procedure. The Monte Carlo procedure was repeated 1000 times, and each repetition resulted in a selection of non-smokers that would be recorded as “misclassified” for that specific iteration. In other words, records were chosen for reclassification in proportion to the predicted smoking likelihood values obtained from the predictive BRFSS model applied to the BC data. While we could know for sure, the procedure attempted to determine those self-reported non-smokers whose smoking status was recorded incorrectly or those who were actually smokers and were unwilling to divulge that fact.

Our random reclassification procedure is akin to the propensity score method, but relies on the Monte Carlo approach to selecting cases with higher likelihoods of smoking, thus accounting for uncertainty in the propensity scores themselves. The primary assumption of the Monte Carlo procedure, as used here, was that the target distribution for the population of misclassified smokers had to match the distribution of self-reported smokers with respect to infant birth weight. This assumption implies that underreporting can happen regardless of how much a mother smokes. To this end, we required the mean, standard deviation, skewness, and kurtosis of infant birth weights from the misclassified records be equal to the mean, standard deviation, skewness, and kurtosis of self-reported smokers for infant birth weights.

Since hospitals are the primary source for birth records in Massachusetts, different data collection procedures at the hospitals could be a possible explanation for underreporting of smoking during pregnancy. All 25 Massachusetts birth hospitals that reported at least 500 self-reported smokers during 1997–2004 were contacted about methods used to complete the parent's birth certificate worksheet. Information was gathered about pre-registration in which parents could complete the birth questionnaire prior to the birth. All hospitals used this practice. In the event that the pre-registration form was not returned prior to delivery, some hospitals used a personal interview to complete the worksheet. Others allowed the mother to complete a worksheet without having a personal interview. We looked at whether this difference in the data collection process related to differences in underreporting by hospital.

### Implementation of the Monte Carlo algorithm

The Surgeon General's 2004 report suggested that smoking during pregnancy results in an average 200 g decrease in infant birth weight [1]. It is important to note that this figure should not be considered a gold standard. It is simply an estimate based on numerous studies. Moreover, since this is a national estimate, there is also no way no way to be certain that birth weights in Massachusetts are affected by a greater or lesser degree from smoking during pregnancy. Our work (shown below) included an estimate of the “effect size” of smoking during pregnancy based on an analysis of key information contained in the Massachusetts birth records. That estimated effect size was 194.6 g.

The Monte Carlo algorithm we designed sought to determine the demographic profile of underreporting if the “true” effect had been something other than 194.6 g. Specifically, we chose to look at 4 different smoking effect sizes for smoking during pregnancy each of which was separated by approximately 3 standard errors from the next closest value. These were: 197.3 g, 200.0 g (the Surgeon General's estimate), 202.7 g, or 205.4 g. It also should be noted that the Surgeon General's report made no separate estimate of an infant weight differential for women who reported smoking prior to pregnancy but subsequently abstained from smoking during pregnancy. Because no separate estimate was made for these “spontaneous quitters”, they were excluded from the remainder of the analysis. Also excluded were cases where the data was suspect (e.g., birth weights more than four standard deviations from the mean).

**Figure 2:**
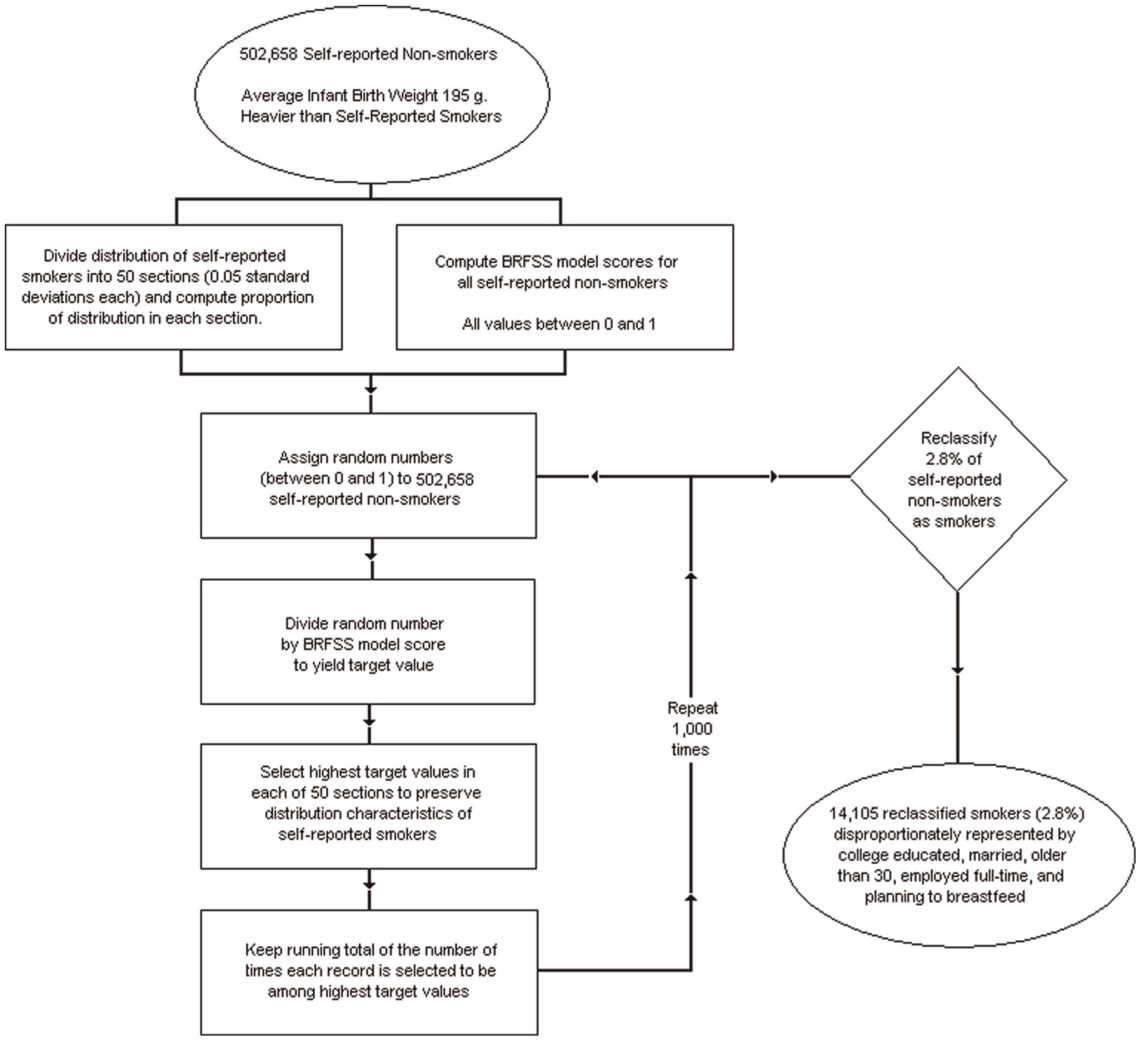
Flow chart of Monte Carlo procedure for reclassifying non-smokers as smokers.

In all four cases, we computed the percent of “non-smokers” that would need to be reclassified to ensure that the difference in average infant birth weights for the population of newly classified smokers and non-smokers would equal 197.3 g, 200.0 g, 202.7 g, and 205.4 g respectively. Assuming the distribution characteristics described above, 1.4% of non-smokers would need to be reclassified as smokers to move the difference in average infant birth weights from 194.6 g to 197.3 g. To move the difference from 194.6 g to the Surgeon General's estimate of 200 g, 2.8% of non-smokers would need to be reclassified as smokers. 4.2% of non-smokers would need to be reclassified as smokers to yield a difference of 202.7 g and 5.6% of non-smokers would need to be reclassified as smokers to yield a difference of 205.4 g. See Figure 2 for a graphic depiction of the reclassification logic using the Surgeon General's estimated unique effect size for smoking during pregnancy (i.e., 200 g).

For each of the target differences (197.3 g, 200.0 g, 202.7 g, and 205.4 g), a total of 1,000 Monte Carlo iterations were completed. As stated above, records were chosen in proportion to the values obtained from the likelihood estimates based on the BRFSS smoker model. In other words, if the estimated likelihood of smoking for one record was 20%, that record was twice as likely to be marked as misclassified as a record with a 10% likelihood estimate. Final Monte Carlo scores were computed by counting the number of times a record was selected and dividing this value by the number of iterations (1000). Finally, demographic and socio-economic patterns of under-reporters were examined by comparing self-reported smoking and the Monte Carlo scores.

For purposes of validation, we also compared the socio-demographic patterns of underreporting during pregnancy estimated by our Monte Carlo reclassification method and those estimated by an existing high-precision method based on cotinine-calibration of smoking self-report [9], [10]. The Dukic et al. estimates used another Massachusetts cohort dataset, the Maternal and Infant Smoking Study of East Boston (MISSEB) [22]. MISSEB study recruited pregnant women at an East Boston neighborhood health clinic between March 1986 and October 1992. Women seeking prenatal care were eligible for the study if they were less than 20 weeks pregnant, spoke English or Spanish, would be at least 19 years of age by the time of delivery and planned to return to the clinic for pediatric care. 1,000 of 1,365 eligible women who came to the clinic were enrolled in the study. This cohort was 52.5% White, 41.4% Hispanic, and 6.1% other race or ethnic status. The recruited women were mostly of low socioeconomic status, and 38.4% of them had less than a high school education. At time of enrollment, the mean age was 26 years, with a range from 18 to 43 years. MISSEB collected information on smoking at each prenatal visit, which consisted of both the self-report as well as urinary and serum cotinine levels. Among the women followed to delivery, 296 (34%) reported smoking sometime during the pregnancy, and 429 (49%) reported smoking at some point in their life. At delivery, average self-reported daily cigarette consumption was 3.5 cigarettes. Although 296 mothers had reported smoking during pregnancy, Dukic identified 330 as smokers based on their cotinine values [10].

## Results

While self-reports of smoking prevalence from birth records may underestimate actual prevalence, studies have shown that self-reports of smoking status on the BRFSS have high validity [24]. Using all female respondents (n = 18,533) between 18 and 44 who participated in the Massachusetts BRFSS between 1997 and 2004, a generalized linear predictive model was developed to predict likelihood of current smoking. The final model had 8 primary demographic and socio-economic variables and 10 interactions. Sensitivity to inclusion/exclusion criteria was examined and the chosen model was found to yield the highest concordance (c = 0.719) or measure of agreement between recorded and estimated pairs based on Kendal's Tau statistic. See Table 1 for details.

The model was then used to predict the likelihood of current smoking for 605,095 mothers with live births in Massachusetts between 1997 and 2004. These model estimates subsequently were used as the primary criteria in the Monte Carlo procedure for marking probable misclassified records.

Since smoking during pregnancy is associated with the lower infant birth weight, any significant misclassification of mothers as non-smokers would reduce the difference in average infant birth weights for self-reported smokers and non-smokers. As stated above, the Surgeon General's 2004 report suggests that the unique effect of smoking during pregnancy is a 200 g average decrease in infant birth weight [1]. In Massachusetts between 1997–2004, the raw average difference in birth weights between self-reported smokers and those who reported not smoking before and during pregnancy was 230 g. However, confounding variables such as the mother's age and nativity, infant sex, maternal weight gain, adequacy of care (Kotelchuck Index) and specific pregnancy risks (e.g., hypertension, gestational diabetes, etc) act to inflate the size of this difference [25]. Once the effect of these confounders was removed, the unique contribution of smoking during pregnancy was 194.6 g.

For a population of 502,658 non-smoking mothers, a 5.4 g difference (200 g–194.6 g) is 6.6 standard errors below the Surgeon General's estimated effect size. Again, it is important to note that the Surgeon General's 200 g estimate of the unique effect of smoking during pregnancy is simply an estimate. In Massachusetts, the best estimate of the unique contribution of smoking during pregnancy based on data from birth records was only 194.6 g. This lower value, however, assumes that the smoking status for all mothers is accurately recorded. As the number of underreports of smoking during pregnancy increases, the average difference in infant birth weights for self-reported smokers and non-smokers is likely to decrease.

Using Monte Carlo procedures, four sets of Monte Carlo scores were computed for the four target estimates for effect size for smoking during pregnancy (i.e., 197.3 g, 200.0 g, 202.7 g, and 205.4 g. Underreporting of smoking during pregnancy varied significantly by demographic and socioeconomic group, but the pattern of significances was virtually identical for the four Monte Carlo runs. Relative increases in the estimates of smoking during pregnancy from the Monte Carlo runs were a function of the percentage of non-smokers reclassified. Fewer reclassifications meant smaller relative increases in smoking prevalence.

Increases were found in the estimated prevalence for every subpopulation tested. Six were significantly higher than the average relative increase for all populations. Compared to the average, there were significant prevalence increases for women who were college-educated, married, aged older than 30 years, employed full-time, and planning to breastfeed. This pattern of disproportionate increases was similar for all four Monte Carlo runs. For a complete description of the demographic profile associated with misclassification using the 200 g effect size, see Table 2.

**Table 2:** Pattern of estimated underreports by demographic category for singleton births in Massachusetts (1997–2004) for 200 g effect size for smoking during pregnancy.

Demographic/Socio-Economic Category	Self-Reported Prevalence	Estimated Prevalence	Proportional Increase (Avg = 1.21)	Higher Relative Increase Compared to Average
WHITE NON-HISPANIC	10.8%	13.3%	1.21	
BLACK NON-HISPANIC	8.6%	11.3%	1.31	
HISPANIC	6.4%	7.8%	1.21	
OTHER RACE	4.8%	7.5%	1.54	Higher (p<.0001)
MARRIED	5.3%	7.5%	1.40	Higher (p<.0001)
SINGLE	22.4%	25.3%	1.13	
NO H.S. DEGREE	24.7%	27.4%	1.11	
H.S. DEGREE	13.4%	16.6%	1.23	
COLLEGE OR MORE	1.3%	2.6%	1.91	Higher (p<.0001)
EMPLOYED	7.7%	10.2%	1.32	Higher (p<.0001)
STUDENT	10.9%	12.4%	1.13	
HOMEMAKER	13.3%	15.4%	1.16	
AGE – UNDER 21	20.6%	22.3%	1.09	
AGE – 21 TO 25	17.0%	19.8%	1.17	
AGE – 26 TO 30	8.8%	11.2%	1.27	
AGE – OVER 30	6.1%	8.3%	1.50	Higher (p<.0001)
BREASTFEED – NO	21.3%	23.9%	1.12	
BREASTFEED – YES	5.8%	8.1%	1.39	Higher (p<.0001)

Underreporting rates also varied significantly by birth facility. Five hospitals located in suburban Boston had rates significantly higher than average. Women who were college-educated, married, older than 30 years, employed full-time, or planning to breastfeed gave birth in higher numbers at these suburban hospitals.

As stated above, we found differences in the methods of data collection at birth hospitals. While all hospitals allowed pre-registration with early submission of the birth questionnaire, there were differences in how the hospitals obtained the completed birth questionnaire if there was no pre-registration. Approximately half used a personal interview while the others simply allowed the mother to complete the questionnaire on her own after arriving at the hospital. Despite these procedural differences, we found no relationship between the rate of likely underreporting and the manner by which self-reports of smoking status were collected at the hospital.

Finally, in order to validate the scoring method described above, we applied it to the data from the MISSEB study. We hypothesized that the demographic pattern of underreporters found in the birth records could be used to predict smoking status, birth weight, and cotinine levels for both self-reported smokers and self-reported non-smokers in MISSEB.

It's important to note that not all variables used in the analysis of birth records were common to MISSEB. Age, race, education, and employment status were significant factors in the prediction of smoking in the Massachusetts birth records. These also were found in MISSEB. Age was recorded in years in both datasets but the form and presentation of the questions about race, education, and employment status were not identical in the two datasets. For example, the birth records used more categories for ethnicity than MISSEB, and so only the common race categories “white” and “black” were included in the model. Similarly, employment status was recorded differently: MISSEB used a binary choice of employed” and “not employed” while the birth records used a text field for “mother's occupation.” Furthermore, between 1997 and 2004, there were over 230,000 unique descriptions of the mother's occupation in the birth records; to match MISSEB, these text fields were recoded by the authors into 2 categories: employed full or part-time and not employed. While education level attained was recorded similarly across the two datasets, some MISSEB records were internally inconsistent. In MISSEB, “grade level attained” and “diploma received” were stored in separate fields. When the values appeared incompatible, we used the value in “diploma received” so we could resolve the incompatibility and match the categories used in the birth records.

Next, a linear regression was performed: the resulting categories of age, race, education, and employment status were used as covariates in the regression of Monte Carlo scores for the 502,658 self-reported non-smoking mothers from the birth records. These linear coefficients were then used to make the estimates of smoking likelihood for the MISSEB dataset. The goal was to determine whether the predicted values of underreporters could accurately distinguish between self-reported smokers and non-smokers in MISSEB. As hypothesized, these smoking likelihood estimates were found to be significantly higher for self-reported smokers than for self-reported non-smokers in MISSEB (t = 9.51, p<.0001). Next, we shifted the focus on self-reported non-smokers in MISSEB only. Here the goal was to determine whether the demographic profile of underreporters from the birth records could accurately predict clinical data for self-reported non-smokers in MISSEB. Specifically, we were interested in infant birth weight and cotinine levels. Here again, the smoking likelihood estimates were important. The predicted values based on the linear coefficients from the birth record regressions correlated significantly with the MISSEB birth weights (r^2^ = 0.08, p<.05) as well as with the log of average cotinine (r^2^ = 0.23, p<.0001).

## Discussion

This study presents a statistical model, based on Massachusetts BRFSS data, which estimates likelihood of current smoking among women. When applied to BC data, the model can be used to estimate population level potential underreporting of smoking during pregnancy and identify demographic and socio-economic characteristics most commonly associated with underreports of smoking. In this analysis, underreports were found disproportionately in women who were college-educated, married, older than 30 years, employed full-time, and planning to breastfeed. That is not to say that prevalence rates for these groups are higher than average. In fact, in many cases, the rates are lower. The proportional increases, however, are significantly higher than for other population subgroups tested.

These findings are consistent with those of the study by Allen [26], which compared self-reports of smoking during pregnancy on the Pregnancy Risk Assessment Monitoring System (PRAMS) survey to BC reports for the same sample of women. The Allen study noted that women who were better educated, older than 25 years, and had health insurance other than Medicaid during pregnancy were likely to report smoking during pregnancy only on confidential questionnaires and not on the BC. In our study, the model predicting likelihood of smoking during pregnancy utilized the BRFSS, a population-based survey, and was not tied to pregnancy as the PRAMS and BC comparison study was. This might serve to minimize misclassification due to maternal underreporting of smoking.

BRFSS self-reports of smoking have been shown to have high validity. Since both data sources in the Allen study relied on self-reports recorded shortly before or after the birth of a child, both sources could be subject to the same information bias. Using the BRFSS, a population in which <4% of women of childbearing age are pregnant at the time of the survey, to develop the model predicting likelihood of smoking should minimize underreporting that might occur during or shortly after pregnancy because of social stigma attached to such behavior.

This model can be used to assess the impact of smoking during pregnancy in other datasets. Since BCs are population-based, they are an attractive data source for studying the impact of smoking during pregnancy. However, given that smoking during pregnancy is likely underreported in BC's, use of these data can lead to underestimates of some effects and to spurious relationships that might be reported as “protective factors” associated with smoking during pregnancy. A predictive model that corrects for potential misclassification of smoking during pregnancy can better quantify the effects of known and heretofore unknown links between smoking and pregnancy outcomes.

There are several limitations to this study. First of all, the BRFSS likely smoker model was developed primarily using responses from women who were not pregnant. More than 96% of the sample were female respondents between 18 and 44 who did not report being pregnant. We looked into using data from the Massachusetts Pregnancy Risk Assessment Monitoring System (PRAMS), but the number of records was much too small to develop a reliable model of smoking likelihood. While it may seem that this population would be markedly different from a population made up entirely of pregnant or recently pregnant women, many of the similar biases affecting respondents to the birth certificate worksheets would likely apply to BRFSS responses. We might argue that the analyses presented in this paper are conservative and represent the lower bound in terms of underreporting during pregnancy. In the end, we concluded that a model based on a large number of records would probably yield more reliable results. Similarly, the BRFSS sample includes an unknown number of women of null parity. Another possible sample group would be pregnant women and recent mothers. Unfortunately, the Massachusetts BRFSS does not routinely include questions about how many children a women has delivered making it impossible to restrict the BRFSS model to this population. When using birth records, there are always questions raised about the accuracy of the information. While some data is surely inaccurate, any bias resulting from these inaccuracies would likely bias results further toward the null hypothesis.

It is important to note that our model was designed to identify demographic and socio-economic characteristics associated with underreporting. The model was not designed to capture other systems-level factors or individual factors that are potential contributors to underreports such as individual respondent characteristics, method and setting of encounter, social desirability of the subject of inquiry, and complexity of the question. These factors play some role in underreporting and more research is needed in order to understand their relative contribution to the issue.

To assess the accuracy of our predictive model, a validation study of the model's predictive ability was conducted using an independent dataset where bioassay calibration of self-report has already been performed [9], [10]. The validation was performed using data from the Maternal and Infant Smoking Study of East Boston (MISSEB), a population-based study of the effects of infant exposure to prenatal maternal smoking and postnatal passive smoking. A high level of concordance was observed.

This validation study has shown that when biological assays are not available, the Monte Carlo reclassification method can provide a more accurate estimate of smoking during pregnancy (or heavy exposure to environmental tobacco smoke) than self-reports alone. This method could thus allow researchers to obtain better estimates of the proportion of births where smoking may impact the health of the mother and the infant.

Similarly, the profile of underreporters can serve as a reminder to health care providers that all socio-demographic groups smoke during pregnancy and that health messages should be delivered without regard for likelihood of smoking within a group. The recent study by Donahue found that nationwide drop in birth weights that could not be completely explained [27]. This drop was statistically larger “in a low-risk subgroup defined by maternal age, race or ethnicity, education, marital status, smoking, gestational weight gain, delivery route, and obstetric care characteristics.” These characteristics are similar to those identified by the Monte Carlo reclassification method lending further support to our results. With a supportive biological validation, the method described here and other similar methods could open the door for affordable adjustments to the estimates of the impact of smoking during pregnancy.

Though the effects of smoking during pregnancy have been understood since the 1960's, and underreports were documented beginning in the late 1980's [1], [28], it appears that research has not yet been able to isolate who might be underreporting or why. While spontaneous quit rates may be high among the demographics associated with underreporting, they may not be as high as self-reports indicate.

In summary, while most pregnant women report their smoking status accurately, the small percentage who do not carry wide-ranging implications for pre-natal healthcare, healthcare costs, government prevalence estimates, and clinical research. This study aimed to estimate the extent of and characteristics associated with underreporting of smoking during pregnancy using a novel approach. The findings highlight substantial differences underreporting among certain demographic subpopulations and across birth facilities. In order for states to obtain a better, more accurate estimate of the true prevalence of smoking during pregnancy, efforts are needed to ensure standard, consistent and effective methods are used by all birth facilities to collect smoking information on the BC, regardless of the demographic characteristics of the patients served. Health systems and providers should be encouraged to ask *every* patient seeking prenatal care whether she smokes, to advise all smokers to quit, and to refer them to services to assist them to quit.
